# Inhibition of melanogenesis and antioxidant properties of *Magnolia grandiflora* L. flower extract

**DOI:** 10.1186/1472-6882-12-72

**Published:** 2012-06-06

**Authors:** Huey-Chun Huang, Wan-Yu Hsieh, Yu-Lin Niu, Tsong-Min Chang

**Affiliations:** 1 Department of Medical Laboratory Science and Biotechnology, China Medical University, No. 91 Hsueh-Shih Road, Taichung, Taiwan, 40402; 2 Department of Applied Cosmetology & Master Program of Cosmetic Science, Hung Kuang University, No. 34, Chung-Chie Rd., Shalu, Taichung City, Taiwan, 43302; 3 Niuer International Skincare Science Research Institute, 7F, No. 618, Ruiguang Rd., Neihu Dist., Taipei, Taiwan

**Keywords:** *Magnolia grandiflora* L, Melanogenesis, Tyrosinase, Melanin, Antioxidant

## Abstract

**Background:**

*Magnolia grandiflora* L. flower is wildly used in Asian as a traditional herbal medication. The purpose of the study was to investigate the antimelanogenic and antioxidant properties of *Magnolia grandiflora* L. flower extract. In the study, the inhibitory effects of *M. grandiflora* L. flower extract on mushroom tyrosinase, B16F10 intracellular tyrosinase activity and melanin content were determined spectrophotometrically. Meanwhile, the antioxidative capacity of the flower extract was also investigated.

**Results:**

Our results revealed that *M. grandiflora* L. flower extract inhibit mushroom tyrosinase activity (IC_50_ =11.1%; v/v), the flower extract also effectively suppressed intracellular tyrosinase activity (IC_50_ = 13.6%; v/v) and decreased the amount of melanin (IC_50_ = 25.6%; v/v) in a dose-dependent manner in B16F10 cells. Protein expression level of tyrosinase and tyrosinase-related protein 1 (TRP-1) were also decreased by the flower extract. Additionally, antioxidant capacities such as ABTS^+^ free radical scavenging activity, reducing capacity and total phenolic content of the flower extract were increased in a dose-dependent pattern.

**Conclusions:**

Our results concluded that *M. grandiflora* L. flower extract decreased the expression of tyrosinase and TRP-1, and then inhibited melanogenesis in B16F10 cells. The flower extract also show antioxidant capacities and depleted cellular reactive oxygen species (ROS). Hence, *M. grandiflora* L. flower extract could be applied as a type of dermatological whitening agent in skin care products.

## Background

Melanin plays an important role in protection the skin against ultraviolet light injury and is responsible for skin color. However, overproduction and accumulation of melanin result in several skin disorders including freckles, melasma, age spots and other hyperpigmentation syndrome [1]. Tyrosinase (monophenol, L-dihydroxyphenylalanine (L-DOPA): oxygen oxidoreductase EC 1.14.18.1) is the key enzyme in the first two steps of melanin biosynthesis, in which L-tyrosine is hydroxylated to L-DOPA (*o*-diphenol product), and L-DOPA is further oxidized into the corresponding *o*-quinone [2]. It has been reported that microphthalmia-associated transcriotion factor (MITF) and other enzymes such as tyrosinase related protein-1 (TRP-1) and tyrosinase related protein-2 (TRP-2) also contribute to the production of melanin [3-5]. Recently, melanogenesis inhibitors have been increasingly applied in skin care cosmetics for the prevention of hyperpigmentation [6]. In addition, melanogenesis is reported to produce hydrogen peroxide (H_2_O_2_) and other reactive oxygen species (ROS) which makes the melanocytes under high-grade oxidative stress. It is well known that ROS play a significant role in the regulation of the melanogenesis, while ROS scavengers and inhibitors of ROS generation may down-regulate UV-induced melanogenesis [7]. Therefore, antioxidants such as ascorbic derivatives and reduced glutathione (GSH) have been applied as inhibitory agents of melanogenesis [8,9].

*Magnolia grandiflora* L. (Magnoliaceae) is widely used as a traditional medicine for the treatment of diarrhea, abdominal diseases, rheumatic arthritis, heart disturbances, high blood pressure, epilepsy, infertility and fever [10]. The aqueous extracts of flowers and leaves have been reported to exhibit cardiovascular effects [11] and was also used as an anticonvulsant in rat [12]. Chemical studies on *M. grandiflora* L. and other plants of the same genus, such as *M*. *officinalis*, have reported the presence of magnolol and honokiol [13,14], which exhibit muscle relaxant activity [15], inhibitory effects on skin tumour promotion [16] and antimicrobial properties [17]. It is interesting to find that *M. grandiflora* L. contains a number of sesquiterpene lactones which possess anti-inflammatory properties and used for treatment of pain [18]. A number of biologically active alkaloids [14], sesquiterpenes [19], phenolic constituents [17], glycosides [20] and other compounds [21] have been isolated from this species. However, scientific studies of the antimelanogenic and antioxidant properties of this medicinal plant are lacking.

The aim of this study was to investigate the inhibitory effects of *M. grandiflora* L. flower extracts on mushroom tyrosinase activity, murine intracellular tyrosinase activity, expression of melanogenesis-related proteins and melanin content in B16F10 melanoma cells, as well as its antioxidant activities.

## Results

### Effect of *M. grandiflora* L. flower extract on B16F10 cell viability

**Figure 1:**
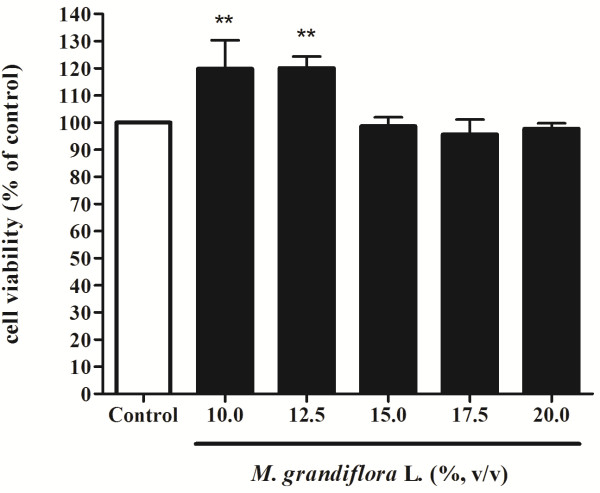
**Effect of *M. grandiflora* L. flower extract on B16F10 cell viability.** Cells were treated with various concentration of flower extract (10, 12.5, 15, 17.5, 20%; v/v) for 24 h and the cell viability was measured by MTT assay. Results are expressed as percentage of cell viability relative to control. Data are presented as mean ± S.D. Values are significantly different by comparison with control. ** *p* < 0.01.

The MTT assay was used to assess the effect of *M. grandiflora* L. flower extract on B16F10 melanoma cells viability. The cells were treated with various concentrations of the flower extract (10, 12.5, 15, 17.5, 20%; v/v) for 24 h and then MTT assay was performed. Results are expressed as percent viability relative to control. After treatment, *M. grandiflora* L. flower extract show no cytotoxic effect on B16F10 cell proliferation (Figure 1).

### Effect of *M. grandiflora* L. flower extract on mushroom tyrosinase activity, B16F10 melanin content and intracellular tyrosinase activity

**Figure 2:**
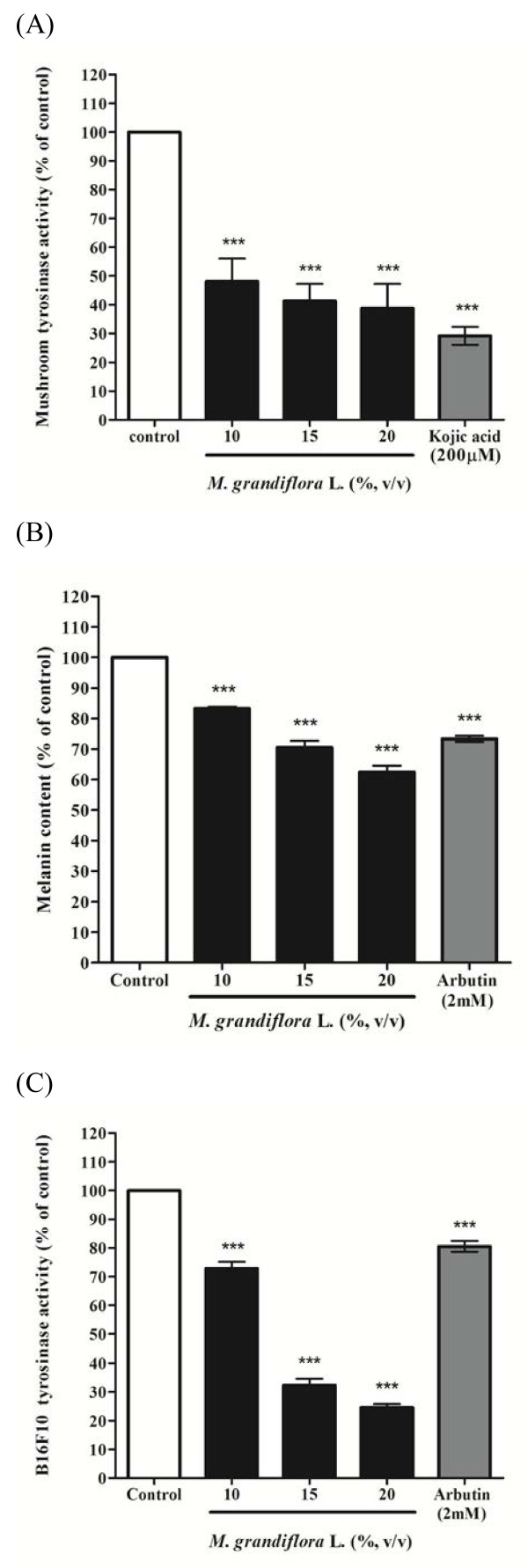
**Inhibitory effect of *M. grandiflora* L. flower extract on mushroom tyrosinase activity, B16F10 melanin content and intracellular tyrosinase actvity. **(**A**) Different concentrations of the flower extract (10, 15, 20%; v/v) or kojic acid (200 μM) was incubated with the same units of mushroom tyrosinase. Following incubation, the amount of dopachrome produced was determined at 490 nm spectrophotometrically. (**B**) & (**C**) B16F10 melanoma cells were stimulated with α-MSH (100 nM) for 24 h, and then the melanin content or intracellular tyrosinase activity were measured after treatment with various concentrations of the flower extract (final concentration 10, 15, 20%; v/v) or arbutin (2.0 mM) for another 24 h. Results are represented as percentages of control, and data are presented as mean ± S.D. for three separate experiments. Values are significantly different by comparison with control. *** *p* < 0.001.

In order to assay the inhibitory effect of the flower extract on mushroom tyrosinase activity, tyrosinase enzyme inhibition experiments were carried out in triplicate. The results indicated that mushroom tyrosinase activity was inhibited by the various concentrations of *M. grandiflora* L. flower extract. The remained tyrosinase activity was 48.14 ± 7.92%, 41.32 ± 5.90% and 38.75 ± 8.5% of control for 10, 15 and 20% (v/v) of the flower extract, respectively. The IC_50_ of the flower extract on mushroom tyrosinase is 11.1% (v/v). Meanwhile, mushroom tyrosinase activity was also inhibited by kojic acid (200 μM) and remained enzyme activity was 29.21 ± 3.12% of that of control (Figure 2A). Hence, *M. grandiflora* L. flower extract may act as a tyrosinase inhibitor.

To determine the antimelanogenic activity of *M. grandiflora* L. flower extract, the inhibitory effect of the flower extract on melanin content in B16F10 melanoma cells was assayed. B16F10 cells were first stimulated with α-MSH (100 nM) for 24 h, and then cultured in the presence of the flower extract at 10, 15 and 20% (v/v) or arbutin (2.0 mM), respectively. Treatment with the flower extract showed a significant inhibitory effect on melanin synthesis in a dose-dependent pattern. The melanin content was represented as percentage of control. After treatment, the melanin content was 83.22 ± 0.64%, 70.49 ± 2.23% and 62.48 ± 2.10% for 10, 15 and 20% (v/v) of the flower extract, respectively (Figure 2B). The IC_50_ of the flower extract on B16F10 melanin content is 25.6% (v/v). Meanwhile, B16F10 cells were treated with arbutin (2.0 mM) as positive standard, and the remained intracellular melanin content was 73.34 ± 1.00% of control for arbutin.

To examine the action mechanism of the inhibitory effect of *M. grandiflora* L. flower extract on melanogenesis more precisely, we assessed intracellular tyrosinase activity in B16F10 melanoma cells. The cells were first stimulated with α-MSH (100 nM) for 24 h, and then cultured with various concentrations of the flower extract (10, 15, 20%; v/v) or arbutin (2.0 mM) for another 24 h. The flower extract significantly inhibited α-MSH-induced tyrosinase activity in a dose-dependent pattern. After these treatments, the remaining intracellular tyrosinase activity was 72.80 ± 2.30%, 32.28 ± 2.35% and 24.61 ± 1.16% for 10, 15 and 20% (v/v) of the flower extract, respectively. The IC_50_ of the flower extract on B16F10 intracellular tyrosinase is 13.6% (v/v). Meanwhile, the intracellular tyrosinase activity was 80.55 ± 1.91% after the cells were treated with arbutin (2.0 mM) (Figure 2C).

### Effect of *M. grandiflora* L. flower extract on expression of melanogenesis-related proteins

**Figure 3:**
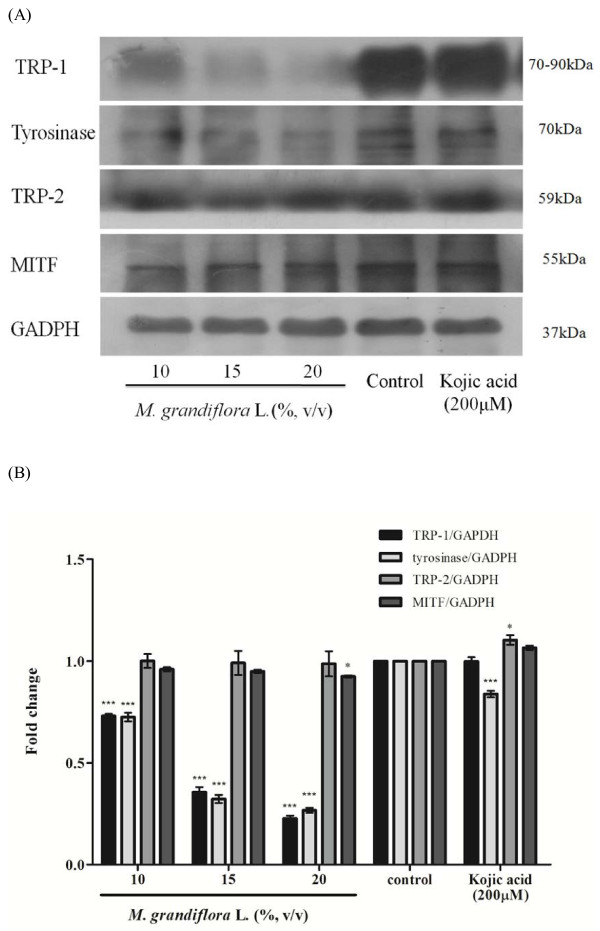
**Effect of *M. grandiflora* L. flower extract on melanogenesis-related proteins expression.** (**A**) B16F10 cells were cultured with α-MSH (100 nM) for 24 h and then treated with various concentration of the flower extract (10, 15, 20%; v/v) or kojic acid (200 μM) for another 24 hr. Then the content of cellular MITF, tyrosinase, TRP-1 and TRP-2 proteins were analyzed by western blotting assay. (**B**) The relative amounts of MITF, tyrosinase, TRP-1 and TRP-2 compared to total GAPDH were calculated and analyzed by Multi Gauge 3.0 software and the values represented the mean of triplicate experiments ± S.D.

To test whether *M. grandiflora* L. flower extract does regulate the expression of melanogenesis-related proteins, B16F10 melanoma cells were treated with α-MSH (100 nM) for 24 h, and then 10, 15, and 20% (v/v) of *M. grandiflora* L. flower extract or kojic acid (200 μM) was added for another 24 h. MITF, tyrosinase, TRP-1, and TRP-2 levels were assayed by Western blot (Figure 3A). It was found that *M. grandiflora* L. flower extract treatment led to a reduced level of tyrosinase and TRP-1 (Figure 3B), but the protein contents of MITF and TRP-2 were not obviously changed after treatment (Figure 3A).

### Antioxidant capacities of *M. grandiflora* L. flower extract

**Figure 4:**
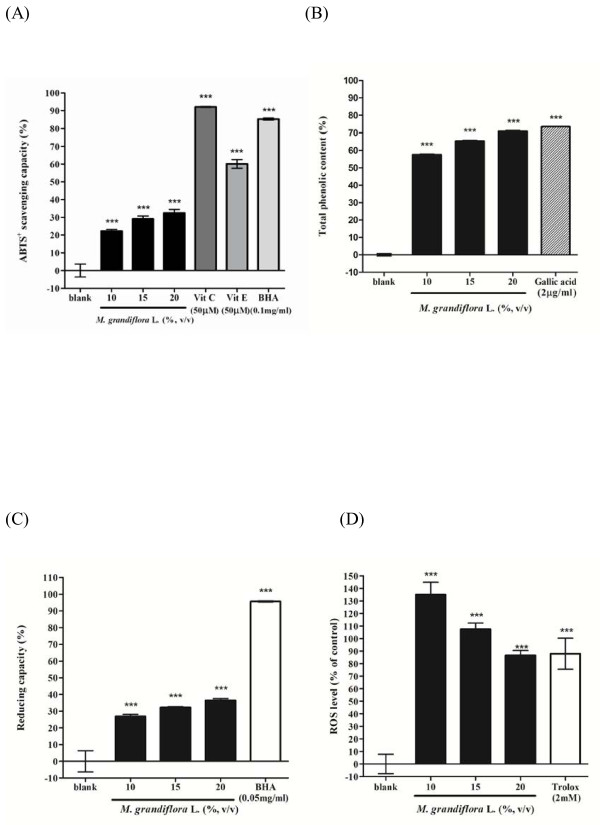
**Antioxidant activities of *M. grandiflora* L. flower extract.** (**A**) ABTS^+^ radical scavenging capacity assay. The flower extract (10, 15, 20%; v/v), vitamin C (50 μM), vitamin E (50 μM) or BHA (0.1 mg/ml) were incubated with ABTS^+^ solution, respectively. (**B**) Determination of total phenolic content. Different concentrations of the *M. grandiflora* flower extract (10, 15, 20%; v/v) and gallic acid (2 μg/ml) were used in the assay. (**C**) Reducing capacity assay. Different concentrations of the flower extract (10, 15, 20%; v/v) or BHA (0.05 mg/ml) were used in the test. (**D**) Determination of ROS content in B16F10 cells. Cells were treated with various concentrations of the flower extract (10, 15, 20%; v/v) or Trolox (2.0 mM) for 24 h and then the ROS content was measured by the DCF-DA assay. Results are represented as percentages of control, and the data are mean ± S.D. for three separate experiments. Values are significantly different by comparison with control. *** *p* < 0.001.

The ABTS^+^ assay was first employed to measure the antioxidant activity of the flower extract of *M. grandiflora* L. Different concentrations of the flower extract (final concentration 10, 15, 20%; v/v), Vitamin C (50 μM), Vitamin E (50 μM) or BHA (0.1 mg/ml) were incubated with ABTS^+^ solution, respectively. The ABTS^+^ scavenging capacity of the flower extract was 22.15 ± 1.07%, 29.06 ± 1.73% and 32.49 ± 1.92% of control for the flower extract at the concentration of 10, 15 and 20% (v/v), respectively. Meanwhile, the ABTS^+^ scavenging capacity of Vitamin C, Vitamin E and BHA was 92.19 ± 0.28%, 60.13 ± 2.48%, 85.29 ± 0.58%, respectively. The results indicated that the flower extract of *M. grandiflora* L. scavenges ABTS^+^ free radical significantly in a dose-dependent manner. However, the flower extract show lower ABTS^+^ radical scavenging capacity than Vitamin C, Vitamin E or BHA do (Figure 4A).

To determine the amount of total phenolic contents of *M. grandiflora L.* flower extract (10, 15, 20%; v/v), gallic acid (2 μg/ml) was used as positive standard. The results in Figure 4B showed that the total phenolic contents in 10, 15 and 20% (v/v) of *M. grandiflora* L. flower extract was 57.50 ± 0.40%, 63.52 ± 0.39%, 70.92 ± 0.43%, respectively. The phenolic content of 20% (v/v) of flower extract was similar to that of gallic acid (73.52 ± 0.19%).

To determine the reducing capacity of *M. grandiflora* L. flower extract, various concentrations of the flower extract (10, 15, 20%, v/v) or BHA (0.05 mg/ml) were tested. The results shown in Figure 4C revealed that higher concentrations of *M. grandiflora* L. flower extract present apparent reducing power. The reducing power of 10, 15, and 20% (v/v) of *M. grandiflora* L. flower extract were 26.89 ± 1.20%, 32.20 ± 0.48% and 36.47 ± 1.08%, respectively.

To confirm the antioxidant capacity of *M. grandiflora* L. flower extract in a cellular environment, evaluation of intracellular ROS levels was done. The concentration of H_2_O_2_ employed was 20 mM. After treatment, the remained intracellular ROS induced by H_2_O_2_ was 86.53 ± 3.60% for 20% (v/v) of the flower extract and 87.89 ± 12.35% for Trolox (2.0 mM) (Figure 4D).

## Discussion

### Inhibition of melanogenesis effects of *M. grandiflora* L. flower extract

The MTT assay is a common colorimetric assay to measure the activity of enzymes that reduce MTT to formazan dyes, giving a purple color. It can also be used to determine the cytotoxicity of potential medicinal agents and toxic materials, since those agents stimulate or inhibit cell viability and growth. The results shown in Figure 1 indicated that even higher concentration of *M. grandiflora* L. flower extract (20%; v/v) also had no cytotoxic effect on B16F10 melanoma cell viability.

Tyrosinase plays an essential role in the first two steps of melanin synthesis pathway. It could convert L-tyrosine to L-DOPA and oxidizes L-DOPA to form dopachrome. Mushroom tyrosinase is widely used as the target enzyme in screening potential inhibitors of melanogenesis. The results shown in Figure 2A indicated that *M. grandiflora* L. flower extract show slightly lower inhibitory effect on mushroom tyrosinase activity than kojic acid does. To elucidate the true inhibitory effect of the flower extract on melanogenesis, B16F10 melanin content and intracellular tyrosinase activity were assayed at the same concentration range. The results shown in Figure 2B indicated that the flower extract from *M. grandiflora* L. present a stronger inhibitory effect on melanin formation in B16F10 cells than arbutin does. The data provided evidence that *M. grandiflora* L. flower extract blocks melanogenesis in B16F10 melanoma cells. The results shown in Figure 2C were in accordance with the results indicated in Figure 2B, which means the flower extract of *M. grandiflora* L. inhibited B16F10 intracellular tyrosinase activity and then decreased the melanin content in a dose-dependent manner. In the experiments, α-MSH was used as a cAMP inducer to stimulate melanin synthesis. It is reported that α-MSH can bind melanocortin 1 receptor (MC1R) and activate adenylate cyclase, which in turn catalyzes ATP to cAMP and increases intracellular cAMP level [22]. In the present study, the results show that *M. grandiflora* L. flower extract inhibited melanogenesis induced by α-MSH mediated intracellular cAMP up-regulation.

TRP-1 and TRP-2 are two enzymes participated in regulation of tyrosinase activity. The results shown in Figure 3A and B indicated that *M. grandiflora* L. flower extract decreased the protein expression levels of both tyrosinase and TRP-1, then inhibited tyrosinase activity and finally decreased melanin content in B16F10 cells. MITF is well known to act as a transcritption factor of tyrosinase. However, in our experiments, the flower extract did not affect MITF expression.

### Antioxidant capacities of *M. grandiflora* L. flower extract

The ABTS^+^ free radical has been widely used as a tool to estimate free radical scavenging activity of antioxidants. Antioxidants, on interaction with ABTS^+^, either transfer electrons or hydrogen atoms to ABTS^+^, thus neutralizing the free radical character [23]. In the present study, the flower extract showed lower free radical scavenging activities as compared to Vitamin C, Vitamin E or BHA.

When assay the total phenolic content of *M. grandiflora* L. flower extract, it was interesting to find that higher concentration of the flower extract (20%, v/v) has similar total phenolic content as gallic acid does. This is probably due to most bioactive compounds such as polyphenols including tannins and flavonoid existed in high polar extracts [24]. Polyphenols are one of the major plant compounds with antioxidant activity. The antioxidant activity of phenolic compounds is reported to be mainly due to their redox properties [25], which can play an important role in adsorbing and neutralizing free radicals, quenching singlet and triplet oxygen, or decomposing peroxides.

In the reducing power assay, it was found that the reducing power of the flower extract was much lower than that of BHA. Even though increases the concentration, the reducing power of the flower extract was still lower than that of BHA.

To confirm the antioxidant capacity of *M. grandiflora* L. flower extract in a cellular environment, evaluation of intracellular ROS levels was done. The principle of the assay is that DCFH-DA diffuses through the cell membrane and is enzymatically hydrolyzed by esterase to DCFH, which reacts with ROS (such as H_2_O_2_) to yield DCF. Rapid increases in DCF indicate oxidation of DCFH by intracellular radicals. The results revealed the flower extract depleted intracellular ROS in a dose-dependent manner. The skin is exposed to UV and environmental oxidizing pollutants and is a preferred target of oxidative stress. It is reported that ultraviolet irradiation induce the formation of ROS in cutaneous tissue provoking toxic changes such as lipid peroxidation and enzyme inactivation [26]. To counteract the oxidative damage, skin is equipped with a network of enzymatic and non-enzymatic antioxidant systems.

It is reported that chronic exposure to solar UV radiation plays a role in the initiation of several skin disorders, including scaling, wrinkling, dryness and mottled pigment abnormalities such as hypopigmentation and hyperpigmentation [27,28]. Therefore, there is an increasing need for new and effective agents which perform photoprotection and skin whitening activities to prevent the above skin disorders. So far, there is no report about the effect of *M. grandiflora* L. flower extract on melanin production. This is the first study to evidence the potential inhibitory effect of *M. grandiflora* L. flower extract on melanogenesis in B16F10 melanoma cells. Besides, the flower extract also show antioxidant capacities, which fit the trend of skin whitening agents show dual functions including anitimelanogenenic and antioxidant activities. For example, several plants such as *Paeonia suffruticosa*[29] and chestnut flower extract [30] havd been reported to show antioxidant and antimelanogenic properties as *M. grandiflora* L. flower extract did. The present results suggested that *M. grandiflora* L. flower extract decreased melanin production may be attributed to inhibition of tyrosinase and TRP-1 or due to its depletion of cellular ROS.

## Conclusions

In the study, *M. grandiflora* L. flower extract show potential dermatological effect against melanin production in B16F10 melanoma cells and present antioxidant capacities. This is the first report about the effect of *M. grandiflora* L. flower extract on melanin production. In the present study, it is found that *M. grandiflora* L. flower extract inhibit melanin synthesis significantly in a dose-dependent pattern. Besides, *M. grandiflora* L. flower extract also expressed antioxidant activities. The results suggested that *M. grandiflora* L. flower extract decreased melanin production may be attributed to its inhibitory action upon the signaling pathway regulating tyrosinase activity or depletion of cellular ROS. We will study the effects of various protein kinase inhibitors on melanogenesis in B16F10 melanoma cells after treatment with *M. grandiflora* L. flower extract in the near future. Certainly, we will also analyze the potential active components in the *M. grandiflora* L. flower extract and elucidate the possible whitening mechanisms.

## Methods

### Chemicals and reagents

Gallic acid, L-ascorbic acid (AA), Folin–Ciocalteau’s phenol reagent, 1,1-diphenyl-2-picrylhydrazyl (DPPH), butylated hydroxyanisole (BHA) and all other chemicals and solvents were obtained from Sigma–Aldrich (St. Louis, MO).

### Preparation of *M. grandiflora* L. flower extract

*M. grandiflora* L. fresh flowers (2 kg) were collected in May 2011 from a farm located at Jianshi township of Hsinchu county in Taiwan. The flowers were air-dried, crushed and added to 800 mL water at room temperature for 48 h to yield 650 mL aqueous extract. The final aqueous extract was stored at 4°C until use.

### Cell culture

B16F10 cells (ATCC CRL-6475; from the BCRC Cell Line Bank, BCRC60031) were cultured in DMEM with 10% fetal bovine serum (FBS; Gibco BRL, Gaithersburg, MD, USA) and penicillin/streptomycin (100 I.U/50 μg/mL) in a humidified atmosphere containing 5% CO_2_ in air at 37°C. All the experiments were performed in triplicate and were repeated 3 times to ensure reproducibility.

### Cell proliferation assay

Cell viability assay of B16F10 was performed using 3-(4, 5-dimethylthiazol-2-yl)-2, 5-diphenyltetrazolium bromide (MTT) [31]. In brief, 1 × 10^4^ cells/well was seeded into a 96-well plate. The cells were exposed to various concentrations of *M. grandiflora* L. flower extract (final concentration 10, 12.5, 15, 17.5, 20%; v/v) for 24 h, the MTT solution was added to the wells. The insoluble derivative of MTT produced by intracellular dehydrogenase was solubilized with ethanol-DMSO (1:1 mixture solution). The absorbance of the wells at 570 nm was read using a microplate reader.

### Assay of mushroom tyrosinase activity

In order to assay the inhibitory action of *M. grandiflora* L. flower extract (final concentration 10, 15, 20%; v/v) on mushroom tyrosinase, dose-dependent inhibition experiments were carried out in triplicate, as described previously, with a minor modification [32]. Briefly, 10 μL of aqueous solution of mushroom tyrosinase (200 units) was added to a 96-well microplate, in a total volume of 200 μL mixture containing 5 mM L-DOPA which is dissolved in 50 mM phosphate buffer (pH 6.8). The assay mixture was incubated at 37°C for 30 min. Following incubation, the amount of dopachrome produced in the reaction mixture was determined spectrophotometrically at 490 nm (OD_490_) in a microplate reader. The inhibition percentage at three doses for each experiment was calculated by the following equation: inhibition percentage of tyrosinase activity (%) = (B-A) ÷ A × 100, where B is the mean of the measured OD_490_ values of the blank control, and A is the mean of the measured OD_490_ values for the *M. grandiflora* L. flower extract treated group.

### Measurement of melanin content

The intracellular melanin content was measured as described by Tsuboi *et al*[33] with some modifications. The cells were treated with α-MSH (100 nM) for 24 h, and then the melanin content was determined after treatment with either *M. grandiflora* L. flower extract (final concentration 10, 15, 20%; v/v) or arbutin (2.0 mM) for a further 24 h. After treatment, the cells were detached by incubation in trypsin/ethylenediaminetetraacetic acid (EDTA). After precipitation, cell pellets containing a known number of cells were solubilized in 1 N NaOH at 60°C for 60 min. The melanin content was assayed by spectrophotometric analysis at an absorbance of 405 nm.

### Assay of B16F10 intracellular tyrosinase activity

Cellular tyrosinase activity was determined as described previously [34] with slight modifications. Briefly, the cells were treated with α-MSH (100 nM) for 24 h, and then intracellular tyrosinase activity was measured after treatment with various concentrations of *M. grandiflora* L. flower extract (final concentration 10, 15, 20%; v/v) or arbutin (2.0 mM) for 24 h. After these treatments, the cells were washed twice with phosphate-buffered saline and homogenized with 50 mM PBS (pH 7.5) buffer containing 1.0 % Triton X-100 and 0.1 mM PMSF. Intracellular tyrosinase activity was monitored as follows: Cell extracts (100 μL) were mixed with freshly prepared L-DOPA solution (0.1% in phosphate-buffered saline) and incubated at 37°C. The absorbance at 490 nm was measured with microplate reader Gen 5^TM^ (BIO-TEK Instrument, Bermont, USA) to monitor the production of dopachrome, corrected for auto-oxidation of L-DOPA.

### ABTS^+^ scavenging capacity assay

The ABTS decolorisation assays were carried out as previously described [35] and it involves the generation of ABTS^+^ chromophore by oxidation of ABTS with potassium persulfate. The ABTS radical cation (ABTS^+^) was produced by reacting 7 mM stock solution of ABTS with 2.45 mM potassium persulfate and allowing the mixture to stand in the dark for at least 6 h before use. Absorbance at 734 nm was measured 10 min after mixing of different concentrations of the *M. grandiflora* L. flower extracts (final concentration 10, 15, 20 %; v/v) with 1 ml of ABTS^+^ solution. The ABTS^+^ scavenging capacity of the filtrate was compared with that of vitamin C (50 μM) and vitamin E (50 μM).

### Determination of total phenolic content

The amount of total phenolics was determined with the Folin–Ciocalteu reagent [36]. First a standard curve was plotted using gallic acid as a standard. Different concentrations of samples were prepared in 80% of methanol. Hundred microliter of sample was dissolved in 500 μl (1/10 dilution) of the Folin–Ciocalteu reagent and 1000 μl of distilled water. The solutions were mixed and incubated at room temperature for 1 min. After 1 min, 1500 μl of 20% sodium carbonate solution was added. The final mixture was shaken and then incubated for 2 h in the dark at room temperature. The absorbance of samples was measuredat 760 nm and gallic acid (2 μg/ml)was used as standard.

### Determination of reducing capacity

The reducing power of the extract was determined according to the method of Oyaizu [37]. Different concentrations of the flower extracts (final concentration 10, 15, 20%; v/v) or BHA (0.05 mg/ml) was mixed with phosphate buffer (2.5 ml, 0.2 M, pH 6.6) and potassium ferricyanide [K_3_Fe(CN)_6_ (2.5 ml, 1% w/v). The mixture was incubated at 50°C for 20 min. A portion (2.5 ml) of trichloroacetic acid (10% w/v) was added to the mixture, which was then centrifuged at 1000g for 10 min. The upper layer of solution (2.5 ml) was mixed with distilled water (2.5 ml) and FeCl_3_ (0.5 ml, 0.1% w/v), and the absorbance was measured at 700 nm in a UV-Vis spectrophotometer. Higher absorbance of the reaction mixture indicated greater reducing power.

### Cellular ROS level determination

B16F10 melanoma cells were cultured in 24-well plates (5 × 10^4^ cells in 1 mL of DMEM medium) and treated with various concentrations of *M. grandiflora* L. flower extract (final concentration 10, 15, 20%; v/v) for 24 h. The cells were then incubated with 24 mM H_2_O_2_ at 37°C for 30 min. After incubation, 2', 7'-dichlorofluorescein diacetate (DCFH-DA) was added to the wells, and the cells were cultured for 30 min. After treatment, the cells were washed with phosphate-buffered saline, and trypsinized by trypsin/EDTA, and the fluorescence intensities of DCF were measured at excitation wavelength 504 nm and emission wavelength 524 nm using a fluorescent reader Fluoroskan Ascent (Thermo Scientific, Vantaa, Finland). The data were analyzed with Ascent software (Thermo Scientific, Vantaa, Finland). Cells with increased ROS content, appeared as a population with a higher fluorescence intensity [38].

### Western Blotting assay

B16F10 cells, treated with *M. grandiflora* L. flower extract (10, 15, 20%; v/v) or kojic acid (200 μM), were washed twice with cold phosphate-buffered saline (PBS), lysed in PBS containing 1% Nonidet P-40, 0.5% sodium deoxycholate, 0.1% sodium dodecyl sulfate (SDS), 5μg/mL of aprotinin, 100 μg/mL of phenylmethylsulfonyl fluoride, 1 μg/mL of pepstatin A, and 1 mM EDTA at 4°C for 20 min, and then disrupted with a needle. Total cell lysates were quantified using a microBCA kit. Proteins (50 μg) were resolved by SDS-polyacrylamide gel electrophoresis and electrophoretically transferred to a polyvinylidene fluoride (PVDF) filter. The nylon filter was blocked for 1 h in 5% fat-free milk in PBST buffer (PBS with 0. 05% Tween-20). After a brief wash in the PBST buffer, the nylon filter was incubated overnight at 4°C with antibodies (anti-microphthalmia-associated transcription (MITF) antibody (1:1,000), anti-TRP1 antibody (1:2,000), anti-TRP2 antibody (1:2,000), anti-GAPDH antibody (1:3,000), and anti-tyrosinase antibody (1:2,000) (Epitomics, Burlingame, CA). Then the primary antibody was removed, and the filter was further washed extensively in PBST buffer. Subsequent incubation with goat anti-mouse antibody (1:10,000) conjugated with horseradish peroxidase was conducted at room temperature for 2 h. The filter was washed extensively in PBST buffer to remove the secondary antibody, and the blot was visualized with ECL reagent. The relative amounts of MITF and tyrosinase, compared to total GAPDH were calculated and analyzed with Multi Gauge 3.0 software (Fuji, Tokyo).

### Statistical analysis

Statistical analysis of the experimental data was performed by one-way ANOVA Tukey test, which was used for comparison of measured data using SPSS 12.0 statistical software (SPSS, Chicago). Differences were considered statistically significant at *p* < 0.05, ** *p* < 0.01 and *** *p* < 0.001.

## Competing interests

All authors are in agreement with the content of the manuscript and the authors do not have any actual or potential conflict of interest, including any financial competing interests, non-financial competing interests, personal or other relationships with other people or organizations within that could inappropriately influence (bias) the work.

## Authors’ contributions

H-CH carried out the tyrosinase-related studies, participated in the enzyme assays and drafted the manuscript. W-YH carried out antioxidant experiments. Y-LN carried out extraction of *M. grandiflora* L. fresh flowers and Western blot. T-MC participated in design and coordination of the study, performed the statistical analysis and drafted the manuscript. All authors read and approved the final manuscript.

## Authors’ information

1. Dr. Huey-Chun Huang is an associate professor in the department of Medical Laboratory Science and Biotechnology in China Medical University.

2. Miss Wan-Yu Hsie is a student in the master program of Cosmetic Science in Hung Kuang University.

3. Mr. Yu-Lin Niu is a lecturer in the department of Applied Cosmetology & Master Program of Cosmetic Science in Hung Kuang University.

*Dr. Tsong-Min Chang is an associate professor in the department of Applied Cosmetology & Master Program of Cosmetic Science in Hung Kuang University. He is also the chairman of the department of Applied Cosmetology and the director of Master Program of Cosmetic Science in Hung Kuang University.

## Pre-publication history

The pre-publication history for this paper can be accessed here:

http://www.biomedcentral.com/1472-6882/12/72/prepub
